# Solid-State Fermentation of *Aspergillus niger* to Optimize Extraction Process of Isoliquiritigenin from *Glycyrrhiza uralensis*

**DOI:** 10.1155/2020/8927858

**Published:** 2020-11-28

**Authors:** Jingwei Hao, Dianwei Li, Yunrong Jing, Lei Zhang, Jiahui Liu, Yubin Ji

**Affiliations:** ^1^Center for Life Science and Enviromental Science, Harbin University of Commerce, Harbin 150076, China; ^2^College of Life Sciences and Technology, Mudanjiang Normal University, Mudanjiang 157011, China

## Abstract

We successfully extracted isoliquiritigenin from *Glycyrrhiza uralensis* via fermentation with *Aspergillus niger* and ultrasonic-assisted extraction. In brief, we used *A. niger* fermentation to culture *G. uralensis* powder, and we optimized some key parameters such as reaction conditions of pH, inoculation concentration of *A. niger*, fermentation time, and solid-liquid ratio. Based on a single-factor experiment, we utilized the response surface methodology (RSM) approach to optimize this extraction procedure. Using the RSM approach, optimized conditions of pH = 3.694, the solid-liquid ratio = 1 : 2.155, and the inoculation concentration of *A. niger* = 1466745 were selected. Optimized conditions resulted in an extraction efficiency of 1.525 mg/g. These results showed that the extraction of isoliquiritigenin was most affected by pH and then the time of fermentation and the solid-liquid ratio. Overall, the developed extraction technique yielded 5 times the amount of isoliquiritigenin when compared to traditional methods.

## 1. Introduction

*Glycyrrhiza uralensis* Fisch (*G. uralensis*) is a traditional medicinal plant used in China for a wide range of uses and a perennial legume herb with thick roots and rhizome. The roots and rhizome of *G. uralensis* are used as a tonic Chinese herbal medicine [1–5]. Qi invigorates the spleen, clears away heat, detoxifies and eliminates phlegm and coughs, and relieves pain and has synergistic effects with a range of drugs. Major components include triterpenoid saponins, flavonoids, and polysaccharides. Flavonoids are the component of *G. uralensis* in the most activity [6].

Isoliquiritigenin is a major dihydroflavonoid extracted from *G. uralensis* [7–9]. The molecular formula of isoliquiritigenin is C_15_H_12_O_4_, and it has a melting point of 198–200°C and forms as yellow needle crystals [10–13]. And the mature technology can be extracted and separated from plants, and its content is often used as an important index for the quality evaluation of *G. uralensis* [2–5]. Isoliquiritigenin has been demonstrated to have anticancer, antioxidation, anti-inflammatory, and antiviral effects and has been used in the treatment of asthma, diabetes, AIDS, and other diseases in humans [14–21].

Microbial fermentation is used to regulate the metabolism of reactants to ensure controlled production of products [22]. In the production of traditional Chinese medicines, microbial fermentation activity can be improved, abundance of components can be altered, and their toxicity can be reduced, thus offering opportunities for research and development of traditional Chinese medicines [23–25]. *Aspergillus niger*, as a large fungus, has the characteristics of vigorous growth, short fermentation cycle, and no toxin production. It is one of the safe strains certified by the Food and Drug Administration (FDA). It can secrete amylase, cellulase, glucosidase, and endoglucanase. Solid-state fermentation of A. niger is an effective method of biotransformation of Chinese medicinal materials by using enzymes produced by A. niger. It has the advantages of improving curative effect, reducing toxicity, and producing new active ingredients [26–28].

Response surface methodology (RSM) is a statistical method used for multivariable problems. RSM aids the design of tests and uses multiple quadratic regression equations to fit functional models between factors and response values [29].

In the current study, *G. uralensis* was used as the raw material to investigate the use of *A. niger* solid-state fermentation on the extraction efficiency of isoliquiritigenin. By modifying four properties of the extraction process, we aimed to develop an efficient process. Properties modified included time of inoculation, pH of fermentation, ratio of *G. uralensis* to extraction fluid liquid, and mass of *A. niger* inoculated. Optimum conditions for the extraction of isoliquiritigenin from *G. uralensis* were established via RSM.

## 2. Materials and Methods

### 2.1. Experimental

#### 2.1.1. Chemicals and Reagents

*G. uralensis* was obtained from Mudanjiang Pharmaceutical Chain Co., Ltd. (Mudanjiang, China), and isoliquiritigenin standard (98% purity) was obtained from the National Institute for the Control of Pharmaceutical and Biological Products (Beijing, China). Anhydrous ethanol was obtained from Nanjing Xingsha Chemical Co., Ltd., methanol from Puyang Wangda Chemical Co., Ltd., and acetonitrile from Jinan Century Tongda Chemical Co., Ltd. All other analytical-grade chemicals and solvents were obtained from Beijing Chemical Reagents Co. (Beijing, China).

#### 2.1.2. Instrumentation and Analytical Conditions

Instrumentation used was as follows: a KQ-400DB ultrasonic cleaner (Shenzhen Keweida Ultrasonic Equipment Co., Ltd.), 98-1-b electronic temperature-regulating heating sleeve (Heze Shengbang Instrument Development Co., Ltd.), tp-213 electronic balance (Sartorius Instrument Equipment Co., Ltd.), and Waters 2695 HPLC (Waters Co., Milford, MA, USA). In addition, a HiQ sil-C18 reversed-phase column (4.6 mm × 250 mm, 5 *μ*m, KYA TECH Corp., Tokyo, Japan) was used for chromatographic separation using a temperature vibration incubator (Shanghai Chuyi Instrument Equipment Co., Ltd).

Acetonitrile-water-acetic acid (32 : 68 : 0.5, v/v/v) was used as the mobile phase for HPLC analyses, with a flow rate of 1.0 mL/min, injection volume of 10 *µ*L, and column temperature of 25°C. Isoliquiritigenin was detected at an absorbance of 350 nm.

The resultant calibration curve had formula *Y* = 3 × 10^7^*X* + 2 × 10^6^ (*R*^2^ = 0.9911), indicating a good linear fit for isoliquiritigenin (*X*: isoliquiritigenin concentration; *Y*: peak area).

### 2.2. Measurement of Water Content of *Glycyrrhiza*

*G. uralensis* was ground into a fine powder using a grinder. Three samples of 1 g *G. uralensis* were weighed and then dried in an oven at 60°C for 24 h to calculate the moisture content. Water content was calculated as

(1) $$\begin{array}{c} Y=\frac{a-b}{a}\times 100\%, \end{array}$$

where *Y* is the moisture content in %; *a* is the initial wet weight; and *b* is the dry weight. Water content of *G. uralensis* was 3.4%.

### 2.3. Activation of *A. niger*: Preparation of the PDA Culture Medium

150 g peeled potatoes were weighed, 750 mL was added, and the mixture was boiled. After boiling, potatoes were ground and filtered, and filter residues were discarded, while hot, filtered mixture was packed into small test tubes at 1/5th total volume. *A. niger* was inoculated in the resulting medium and cultured at 37°C in the incubator for 4 days.

### 2.4. Ultrasonic-Assisted Extraction of *A. niger* following Fermentation

4 g of *G. uralensis* powder was weighed into a 100 mL conical flask, and distilled water was added at a ratio of solid to liquid and stirred to prepare the sterilized fermentation medium. To investigate the impact of pH on the extraction efficiency of isoliquiritigenin from *G. uralensis*, pH values of 3, 4, 5, 6, and 7 were selected. To investigate the effect of fermentation time on the extraction efficiency of isoliquiritigenin from *G. uralensis*, cultures were maintained for 2, 4, 6, and 8 days, respectively. To investigate the effect of material-to-liquid ratios on the extraction efficiency of isoliquiritigenin from *G. uralensis*, ratios of 1 : 2, 1 : 3, 1 : 4, 1 : 5, and 1 : 6 were selected. To investigate the effect of the inoculation count of *A. niger* on the extraction rate of isoliquiritigenin from *G. uralensis*, inoculation counts of 1 × 10^5^, 5 × 10^5^, 1 × 10^6^, 2 × 10^6^, 3 × 10^6^, 4 × 10^6^, and 5 × 10^6^*A. niger* were investigated. Extraction efficiencies are expressed as the observed values of the target analytes.

### 2.5. Method of Extraction of Isoliquiritigenin following Fermentation

100 mL of 75% ethanol was added to the fermented medium, mixed, and extracted using an ultrasonic water bath at 80°C for 0.5 h. Then, filtrates were collected, and volumes were assessed to determine extraction rates.

### 2.6. Reference and Conventional Extraction Methods

Ethanol reflux extraction: 4 g of *G. uralensis* was weighed into a 250 mL round bottom flask, 100 mL of 75% ethanol solution was added, the solution was mixed, and reflux extraction was completed at 80°C in a water bath for 2 h. The resulting mixture was filtered while hot, and the filtrate was collected.

Ultrasonic extraction: 4 g of *G. uralensis* was weighed into a 250 mL round bottom flask, 100 mL of 75% ethanol solution was added, and the solution was mixed and extracted at 80°C in a water bath for 0.5 h using a sonicator. The resulting mixture was filtered while hot, and the filtrate was collected.

### 2.7. Optimization of Isoliquiritigenin Extraction by RSM

RSM was employed to optimize fermentation conditions using Box–Behnken data processing software (Design-Expert 7.0, Delaware, USA). Using the single-factor investigation approach, pH, inoculation concentration of *A. niger*, and solid-liquid ratio were used as the independent variables and extraction efficiency of isoliquiritigenin as the dependent variable.

### 2.8. Statistical Analyses

To indicate the extraction efficiency of isoliquiritigenin, one-way ANOVA was used to determine the significant differences between experiments with different conditions. All statistical significances were accepted when *α* < 0.05. The results of HPLC analysis were expressed as means of extraction efficiency ± SD. Data analyses were conducted in SPSS 22.0 software.

## 3. Results and Discussion

### 3.1. Single-Factor Experimental Design

#### 3.1.1. Impact of Fermentation pH on UAE-Mediated Isoliquiritigenin Extraction from *G. uralensis*

**Figure 1:**
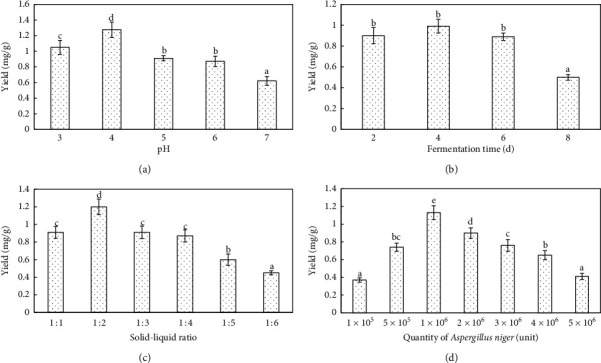
The extraction condition optimization. The following extraction parameters were utilized: (a) 0.4 g of the dried sample was combined with 75% ethanol aqueous solution. The inoculation count of *A. niger* was 2 × 10^6^, solid-1iquid ratio was 1 : 4, fermentation time was 6 days, and inoculation temperature was 36°C. (b) 0.4 g of the dried sample was combined with 75% ethanol aqueous solution. The inoculation count of *A. niger* was 2 × 10^6^, the solid-1iquid ratio for this extraction was 1 : 4, pH was 5, and the temperature was 36°C. (c) 0.4 g of the dried sample was combined with 75% ethanol aqueous solution. Inoculation count of *A. niger* was 2 × 10^6^, fermentation time was 6 days, pH was 5, and temperature was set to 36°C. (d) 0.4 g of the dried sample was combined with 75% ethanol aqueous solution, solid-1iquid ratio was 1 : 4, time of fermentation was 6 days, pH was 5, and temperature was 36°C. Different lowercase letters on the bars represent significant differences (*P* < 0.05) between treatments.

The effect of fermentation pH on the extraction of isoliquiritigenin from *G. uralensis* is presented in Figure 1(a). When pH was 4, the rate of the extraction of isoliquiritigenin was relatively high and differed with pH. As pH of fermentation increased, the extraction efficiency decreased. There were significant differences between different pH (*P* < 0.05). These results were consistent with the previous work which has demonstrated that optimal pH of proteases of *A. niger* is approximately 4.

#### 3.1.2. Impact of Fermentation Time on Isoliquiritigenin Yield

The effect of fermentation time on the extraction rate of isoliquiritigenin from *G. uralensis* was investigated. As presented in Figure 1(b), there were significant differences between different fermentation times (*P* < 0.05). First, with the increase of fermentation time, the extraction rate of isoliquiritigenin increased. After 4 days of fermentation, the extraction of isoliquiritigenin was the highest. Subsequently, the extraction rate of isoliquiritigenin decreased with the increase of days. During the early stages of fermentation, growth and metabolism of *A. niger* would rely on available substrates such as the cell wall, thus promoting the release of isoliquiritigenin. Therefore, the concentration of isoliquiritigenin would have initially increased and then decreased as it might have been used as a substrate, thus decreasing its abundance.

#### 3.1.3. Impact of Solid-Liquid Ratios on Isoliquiritigenin Yield

The extraction of isoliquiritigenin from *G. uralensis* was significantly impacted by the ratio of solid to liquid. As presented in Figure 1(c), the rate of extraction of isoliquiritigenin tended to decrease with increasing the ratios of solid to liquid. The results showed that there were significant differences among different solid-liquid ratios (*P* < 0.05). At the solid-1iquid ratio = 1 : 2, the rate of extraction of isoliquiritigenin was the highest, relatively.

#### 3.1.4. Impact of Inoculation Concentration of *A. niger* on the Extraction of Isoliquiritigenin from *G. uralensis*

As presented in Figure 1(d), the extraction of isoliquiritigenin increased linearly with the increasing concentrations of *A. niger*. There were significant differences between different quantities of *A. niger* (*P* < 0.05). When the inoculation concentration was equal to 1 × 10^6^, the extraction of isoliquiritigenin was the highest.

### 3.2. RSM Optimization of Fermentation Conditions

To further investigate interactions among fermentation conditions and optimize the extraction of isoliquiritigenin, RSM was applied. Experimental randomization was conducted as detailed in Table 1 in an effort to maximize the impact of unexplained variability on extraction efficiency. In total, we conducted 17 tests, with 5 replicates (runs 2, 7, 8, 12, and 15, Table 1), to estimate the pure error sum of squares.

The predicted *R*^2^ value of 0.8235 was reasonably consistent with the adjusted *R*^2^ value of 0.9482, and the ratio of precision was 18.461, thus indicating adequate precision (Table 2). Furthermore, our model had high *F* values and low *P* values (*P* < 0.0001) for two calculated responses. The *F* value of 33.51 implies a 0.01% probability that is due to a random chance. For the result of RSM, any “Prob > *F*” values <0.0500 are significant, whereas values > 0.1 were not considered significant. Based on these criteria, the terms *B*, *C*, *AB*, *B*^2^, and *C*^2^ were considered significant (Table 3).

The “predicted *R*-squared” of 0.8235 is in reasonable agreement with the “adjusted *R*-squared” of 0.9482, with a difference less than 0.2. “Adeq precision” measures the signal-to-noise ratio, where a ratio greater than 4 is desirable. The observed ratio of 18.461 indicates an adequate signal, suggesting that the model can be used to model the design space.

Results of the RSM analysis suggest that three independent variables were related as identified using the second-order polynomial equation: yield (mg/g) = 1.49–0.016*A* + 0.054*B* + 0.20*C* − 0.095*AB* + 0.0025*AC* − 0.062*BC* − 0.049*A*^2^ − 0.21*B*^2^ − 0.33*C*^2^.

**Figure 2:**
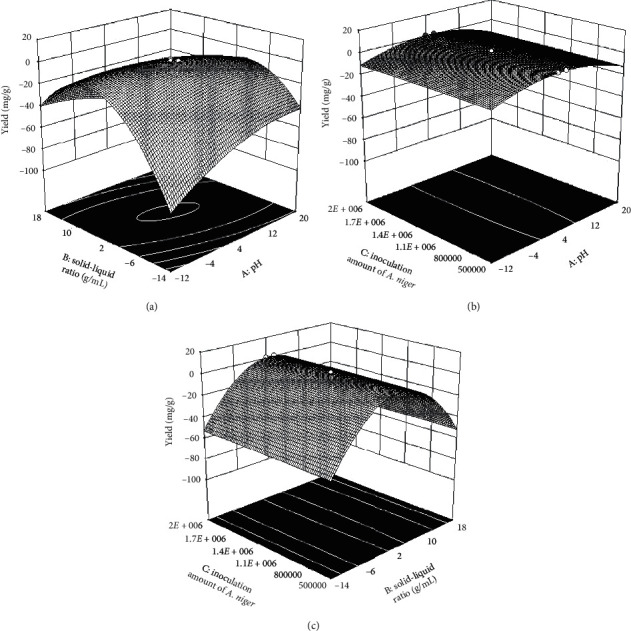
Response surface plots for the effect of variables on the average extraction efficiency of the target analyte. (a) Interaction of pH and solid-liquid ratio. (b) Interaction of pH and the inoculation concentration of *A. niger*. (c) Interaction of the inoculation concentration of *A. niger* and solid-liquid ratio.

The response surfaces for the impact of the independent variables on the average extraction efficiency of isoliquiritigenin are shown in Figure 2. Figures 2(a)–2(c) show the interaction of pH, solid-liquid ratio, and inoculation concentration of *A. niger*. Results of the analysis resulted in point predictions of pH = 3.694, the solid-liquid ratio = 1 : 2.155, and the inoculation concentration of *A. nige*r = 1466745. Overall, the extraction efficiency was 1.525 mg/g.

### 3.3. Verification Tests

Verification tests were conducted three times using point-prediction RSM conditions (pH = 3.694, solid-liquid ratio = 1 : 2.155, and inoculation concentration of *A. niger* = 1466745). The confirmatory analysis yielded a yield efficiency of 1.49 ± 0.035 mg/g.

### 3.4. Comparison of Extraction Methods

Two extraction approaches were used to compare extraction efficiencies: solid-state fermentation of *A. niger* and ultrasonic-assisted extraction of isoliquiritigenin from *G. uralensis*. Extraction process was as follows: 4 g of the dried sample was mixed with 75% ethanol solution and refluxed for 2 h at a specific ratio of solution to raw material of 100 mL. The overall yield was 0.167 mg/g. The condition of the other method was similar to that outlined above, but ultrasonic extraction was used. Ultrasonic extraction methods destroy the external structure of the medicinal material at a specific frequency; therefore, the solvent can fully penetrate the medicinal material, resulting in a shortened extraction time and improved extraction rate. In the present experiment, the extraction rate was 0.31 mg/g.

## 4. Conclusions

In the current study, the fermentation of *A. niger* and ultrasonic-assisted extraction of isoliquiritigenin from *G. uralensis* were studied. A Box–Behnken design was employed to optimize extraction parameters. As demonstrated, the developed extraction process compared favorably (5x) with traditional extraction methods. To investigate the influence of extraction parameters, the RSM approach was utilized as employed in Box–Behnken software to optimize extraction procedures. In the analysis, pH, inoculation concentration of *A. niger*, and solid-liquid ratios were used as independent variables and extraction rate of isoliquiritigenin as the dependent variable. Optimized conditions were pH = 3.694, solid-liquid ratio = 1 : 2.155, and inoculation concentration of *A. niger* = 1466745 unit as the extraction efficiency of 1.525 mg/g was reached.

Because isoliquiritigenin in *G. uralensis* is present as combined and free portions, traditional solvent HRE methods of extraction only extract isoliquiritigenin from *G. uralensis*. The binding of isoliquiritigenin in *G. uralensis* is via aglycone; therefore, growth and metabolism of *A. niger* can release *β*-glucosidase, which can hydrolyze binding isoliquiritigenin into free isoliquiritigenin, thus increasing the rates of extraction. Results showed that fermentation time, solid-liquid ratio, pH, inoculation concentration of *A. niger*, and other factors all influence the rate of extraction of isoliquiritigenin. pH influenced the rate of extraction of isoliquiritigenin from *G. uralensis* most, likely due to the fact that pH impacts growth and fermentation of *A. niger*. During growth and metabolism of *A. niger*, a number of enzymes are produced, and some enzymatic reactions occur. The cell wall of the plant tissue is mainly composed of cellulose, hemicellulose, pectin, and other macromolecules. Some specific enzymatic reactions can hydrolyze cellulose, pectin, and other macromolecules; therefore, components of interest are released and more easily extracted. During the second day of fermentation, mycelium of *A. niger* grew and began to produce spores. The enzymatic activity of secretions would have increased rapidly, and correspondingly, dissolution of isoliquiritigenin reached the maximum value. As the time increased, some cells might have begun to die or degrade extracted products.

## Figures and Tables

**Table 1:** Box–Behnken experimental design.

Run	Factor *A* (pH)	Factor *C* (solid-liquid ratio) (g/mL)	Factor *B* (inoculation amount of *A. niger*)
1	5	3	1.25 × 10^6^
2	4	2	1.25 × 106
3	4	1	5 × 10^5^
4	3	1	1.25 × 10^6^
5	4	3	5 × 10^5^
6	5	2	2 × 10^6^
7	4	2	1.25 × 10^6^
8	4	2	1.25 × 10^6^
9	3	2	2 × 10^6^
10	5	2	5 × 10^6^
11	4	3	2 × 10^6^
12	4	2	1.25 × 10^6^
13	3	3	1.25 × 10^6^
14	4	1	2 × 10^6^
15	4	2	1.25 × 10^6^
16	5	1	1.25 × 10^6^
17	3	2	5 × 10^5^

**Table 2:** Credibility analysis of regression equations.

Index marka	Extraction efficiency of lignans
Std. dev.	0.061
Mean	1.21
C.V. %	5.02
PRESS	0.2
*R*-squared	0.9773
Adjusted *R*-squared	0.9482
Predicted *R*-squared	0.8235
Adeq precision	18.461

**Table 3:** Test of significance for the regression coefficient.

Source	Sum of squares	*Df*	Mean square	*F* value	*P* value
Model	1.11	9	0.12	33.51	<0.0001
*A*-pH	2.112 × 10^−3^	1	2.112 × 10^−3^	0.57	0.4739
*B*-solid-liquid ratio	0.023	1	0.023	6.26	0.0408
*C*-inoculation amount of *A. niger*	0.33	1	0.33	88.91	<0.0001
*AB*	0.036	1	0.036	9.78	0.0167
*AC*	2.500 × 10^−5^	1	2.500 × 10^−5^	6.776 × 10^−3^	0.9367
*BC*	0.016	1	0.016	4.23	0.0786
*A* ^2^	0.010	1	0.010	2.78	0.1391
*B* ^2^	0.18	1	0.18	50.04	0.0002
*C* ^2^	0.46	1	0.46	125.71	<0.0001
Residual	0.026	7	3.690 × 10^−3^		
Lack of fit	0.011	3	3.708 × 10^−3^	1.01	0.4759
Pure error	0.015	4	3.676 × 10^−3^		
Cor total	1.14	16			

## Data Availability

The data used to support the findings of this study are included within the article.
